# Bilateral renal abscess fusing with the psoas on the right: A case report

**DOI:** 10.1016/j.eucr.2021.101951

**Published:** 2021-11-19

**Authors:** Ramzi Mejri, Khaireddine Mrad Dali, Kays Chaker, Bibi Mokhtar, Sami Ben Rhouma, Yassine Nouira

**Affiliations:** a Departement of Urology, Hospital Mongi Slim La Marsa, Tunisia; b Departement of Urology, La Rabta Hospital, Tunisia

**Keywords:** Abscess, Bilateral, Psoas, Percutaneous drainage

## Abstract

Renal abscess is a medical and surgical urological emergency whose diagnosis has been improved by modern imaging.

It often poses a problem of therapeutic management between antibiotic therapy or the association of a drainage. Most abscesses are unilateral, the bilateral nature of the abscessed lesions suggests a hematogenous diffusion.

We report a case of a bilateral renal abscess fusing to the psoas muscle on the right that progressed well with antibiotic treatment and percutaneous drainage.

## 1 Introduction

Renal abscess is a urological emergency whose diagnosis has benefited from the contribution of modern imaging.

It is one of the most serious infectious complications of acute pyelonephritis and acute bacterial and focal nephritis, often occurring on a fragile terrain.

In addition to the diagnostic problem, it poses a problem of therapeutic approach between antibiotic therapy or associated with percutaneous or surgical drainage.

We report the case of a bilateral renal abscess that progressed well under antibiotic treatment and percutaneous drainage.

## 2 Case report

A 19-year-old man presented to our urological surgery unit complaining of bilateral lumbar pain associated with a week-long fever. The patient also reported transit disorders for 5 days, anorexia, asthenia, malaise and weight loss not quantified. The patient's medical history included well-controlled diabetes mellitus and a report of a neglected cat bite one month ago. His clinical examination shows a defense of the right lumbar fossa and a sensitivity of the left lumbar fossa. The rectal examination found a flat and painless prostate. However, the urine was clear and no infectious portal of entry (dental, skin) was identified.

**Fig. 1:**
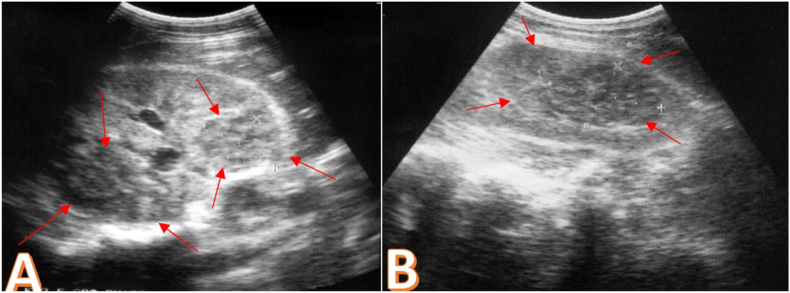
Renal ultrasound: multiple heterogeneous uncollected echogenic formations. A. Right kidney. B. Left kidney.

**Fig. 2:**
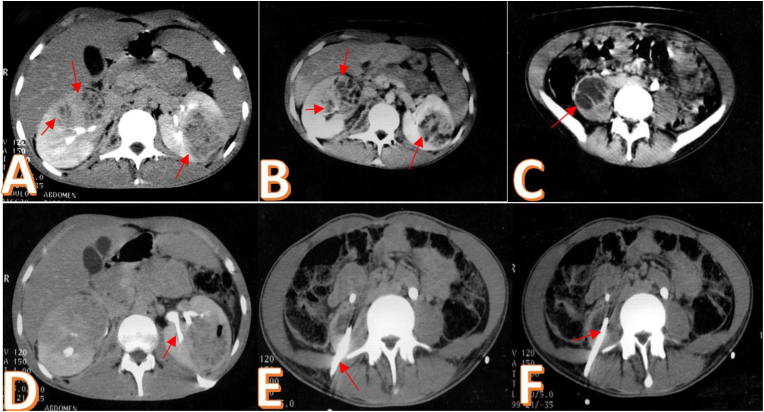
Computed tomography: A, Hypodense septate formations of both kidneys. C. Abscessed collection of the right psoas muscle. D. Drain in the left renal collection. E, F. Drain at the level of the right psoas collection.

**Fig. 3:**
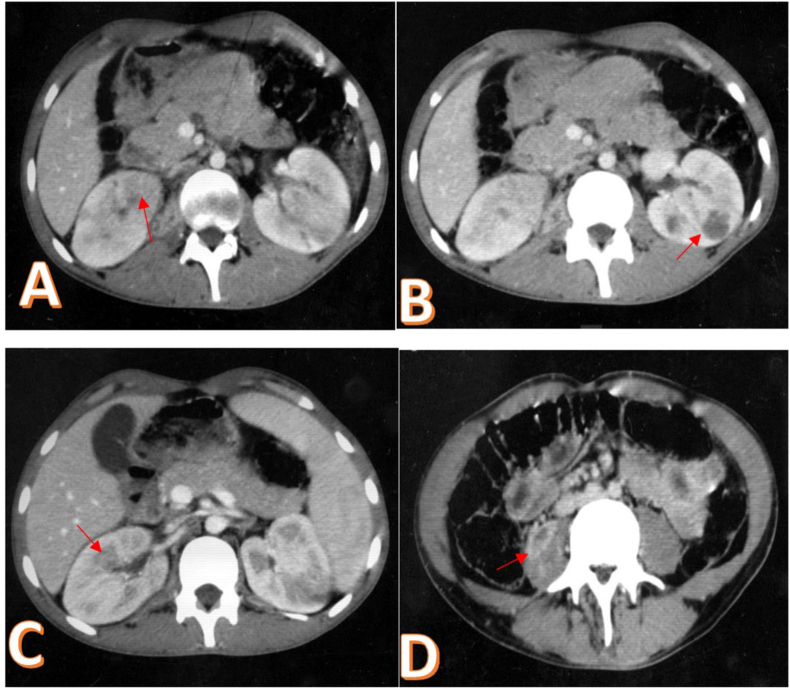
Control CT scan: Clear regression of collections on both sides.

Her complete blood count showed hyperleukocytosis at 18600/mm^3^, a moderate inflammatory syndrome (CRP 68 mg/l) and a normal coagulation panel. Cytobacteriological analysis of the urine was normal as well as his renal function. The blood culture series also came back negative. In view of this clinical presentation of febrile low back pain and in order to support the diagnosis, a renal ultrasound was performed. It showed multiple heterogeneous uncollected echogenic formations of both kidneys with non-dilated excretory cavities and no urinary lithiasis (Fig. 1). Computed tomography revealed several hypodense septate formations of both kidneys associated with an abscessed collection of the right psoas muscle measuring 35mm in diameter (Fig. 2 A, B, C). The radiological data were in favor with the diagnosis of bilateral renal abscess fusing to the psoas on the right. A bilateral drainage puncture under Computed Tomography control was performed urgently. One drain in the large left renal collection, and another in the psoas collection (Fig. 2 D, E, F). Drainage resulted in 200 cc of frank pus allowing isolation of multiresistant *Staphylococcus aureus*. Antibiotic therapy adapted to the antibiogram was instituted combining imipenem and aminoside. The patient also received analgesics, preventive anticoagulation and effective vascular filling. The clinical evolution was marked by a drop in temperature after 7 days and a gradual improvement of the general state with a relaxation of the lower back. The biological assessment was normalized after two weeks. The control CT scan performed after three weeks of drainage showed a clear regression of the lesions on both sides (Fig. 3).

## 3 Discussion

Abscesses of the renal cortex develop primitively by hematogenous diffusion from other infectious sites.

The most frequent germ involved is staphylococcus. Other germs can be incriminated: enterobacteria, pseudomonas, klebsiella and Proteus.^1^

Our patient had bilateral staphylococcal renal abscesses of probably hematogenous origin. The spread of staphylococci to the renal cortex from a cutaneous site via the bloodstream remains the most likely hypothesis, despite a negative etiological investigation. Most abscesses are unilateral (97%), with single lesions (77%) occurring in the right kidney (63%).^2^ Due to high blood flow and lower interstitial pressure, abscess formation is more favored in the renal cortex than in the medulla.

Psoas abscesses usually present as primary or secondary depending on the presence or absence of an underlying condition. The primary form is likely to be caused by hematogenous spread from a distant infectious focus and favored by particular underlying conditions (diabetes, intravenous drug abuse, renal failure and immunosuppression).^3^ The secondary form is essentially caused by the contiguous spread of an infectious process from adjacent organs such as the kidney in most cases. This type of secondary involvement of the psoas muscle has been reported in our presentation.

On ultrasonography, the abscess appears as a thick-walled, rounded mass of regular thickness with a variable echogenic center.^4^

The Computed Tomography scan shows one or more rounded lesions, well limited and spontaneously hypodense.^4^

Ultrasound and Computed Tomography scans are currently used to diagnose kidney abscesses in 92–96% of cases and to guide a therapeutic procedure.^4^

These lesions often pose a problem of differential diagnosis with renal tumor.

The clinical presentation (fever, tenderness or defensiveness of the lumbar fossa) and the biological infectious syndrome guide the diagnosis.

Bilateral renal abscesses are a medical and surgical emergency that can be potentially life-threatening, especially in fragile patients (diabetes, corticosteroids or immunocompromised patients).

The treatment is essentially double. It includes broad-spectrum systemic antibacterial treatment and percutaneous or surgical drainage of the abscess. Percutaneous drainage is a minimally invasive procedure that can be performed under local anesthesia and that allows the abscess to be evacuated as effectively as surgical drainage with less parenchymal damage.

It also allows a bacteriological sample to be taken to isolate the germ and study its sensitivity to antibiotics.

Drainage is performed under radiological control (ultrasound or CT scan) and is indicated for collections of more than 3 cm.^5^ If percutaneous drainage fails, surgical drainage is required.

Treatment should be as conservative as possible. The typical indication for percutaneous drainage is bilateral forms. Treatment of the initial infection and an etiological assessment to look for underlying pathologies must be performed without delay.

## 4 Conclusion

Percutaneous drainage of renal abscesses is easy to perform, minimally invasive and well tolerated by the patient.

It allows, in association with an adapted antibiotic therapy, an effective and definitive treatment.

It is the standard indication for the treatment of multiple or bilateral renal abscesses in order to preserve the maximum nephron capital.

## Authors contributions

R. Mejri: participated in the writing of the manuscript.

K. Mrad Dali: participated in the writing of the manuscript.

K.Chaker: participated in the writing of the manuscript.

M.Bibi: participated in the writing of the manuscript.

S. Ben Rhouma: participated in the writing of the manuscript and its correction.

Y. Nouira: participated in the writing of the manuscript and its correction.

## Declaration of competing interest

The authors declare that there are no conflicts of interest regarding the publication of this article.
